# Does total hip arthroplasty via the direct anterior approach using dual mobility increase leg length discrepancy compared with single mobility?

**DOI:** 10.1186/s42836-020-00060-6

**Published:** 2021-01-31

**Authors:** Seiya Ishii, Yasuhiro Homma, Tomonori Baba, Yuta Jinnai, Xu Zhuang, Hiroki Tanabe, Sammy Banno, Mikio Matsumoto, Taiji Watari, Yu Ozaki, Hironori Ochi, Kazuo Kaneko

**Affiliations:** Department of Orthopaedic Surgery, Juntendo University, 1-1-2, Hongo, Bunkyo-ku, Tokyo, 113-0033 Japan

**Keywords:** Leg length discrepancy, Direct anterior approach, Dual mobility cup

## Abstract

**Background:**

Total hip arthroplasty (THA) via the direct anterior approach (DAA) using dual mobility cup (DMC) is considered to effectively prevent postoperative dislocation. However, the dislocation and reduction procedure using a trial implant during the surgery is difficult because of high soft tissue tension. Thereby, leg length discrepancy (LLD) is difficult to assess when using DM via the DAA.

**Purpose:**

To compare the LLD between cases using conventional SM and those using DMC in THA via the DAA with fluoroscopy.

**Patients and methods:**

We retrospectively investigated 34 hips treated with DMC (DMC-DAA group) and 31 hips treated with SM (SM-DAA group). The LLD was defined as the difference in the distance from the teardrop to the medial-most point of the lesser trochanter between the operative and nonoperative sides at immediate postoperative X-ray.

**Results:**

The mean LLD in the DMC-DAA group and SM-DAA group was 0.68 ± 7.7 mm and 0.80 ± 5.5, respectively, with no significant difference. The absolute value of the LLD in the DMC-DAA group and SM-DAA group was 6.3 ± 4.4 mm and 5.9 ± 5.5, respectively, with no significant difference.

**Conclusion:**

Despite the difficulty in assessment of the LLD during THA via the DAA using DMC, this technique does not increase the LLD compared with the use of SM.

**Level of evidence:**

III, matched case-control study.

## Introduction

Postoperative dislocation is a serious problem in both primary and revision total hip arthroplasty (THA). Recurrent dislocation results in residual pain and contracture of the hip joint, decreasing the patient’s functional score and quality of life [1]. Although optimal cup position angle range was defined by Lewineck in 1978 and has long been an absolute principle for hip surgeons [2], Matthew mentioned its validity was low and that the majority of the dislocation cases were within the “safe zone” [3].

The dual mobility cup (DMC) was developed by Bousquet in 1974 and is frequently used because of its resistance to postoperative dislocation [4]. The DMC is characterized by dual articulation of the cup, increased range of motion before impingement and dislocation. Guyen et al. [5] reported that the DMC increased flexion by 30.5° compared with the conventional 22.2-mm-diameter femoral head implant. Additionally, a systematic review revealed the superiority of the DMC [6]. The dislocation rate at a mean of 6 years after primary THA was 0.9% in the DMC group and 6.8% in the conventional single mobility (SM) cup group. Moreover, the dislocation rate at a mean of 4.1 years after revision surgery was 2.2% in the DMC group and 7.1% in the traditional revision group.

The surgical approaches to THA also influence the dislocation rate [7, 8]. The direct anterior approach (DAA) is receiving mounting attention because it reduces risk of dislocation and provides early postoperative function [9, 10]. Although the dislocation rate of the DAA is relatively lower than those of other approaches, dislocation still occurs.

A few researchers have described the combination of the DAA and DMC. In one report, Homma et al. [11] described the safety and dislocation resistance of this method, especially in patients with a high risk of postoperative dislocation. THA for femoral neck fractures has a higher risk of postoperative dislocation than THA for osteoarthritis [12], and some authors have indicated that THA using DMC via the DAA is a desirable choice for femoral neck fractures [13]. Batailler et al. [14] also reported non-inferiority of THA using DMC via the DAA compared with DAA using SM in terms of the higher complication rate and non-optimal implant position.

However, we found the dislocation and reduction were difficult during THA with DMC via the DAA using a trial implant because of high soft tissue tension and large polyethylene head. In their editorial, Lustig et al. [15] also mentioned that the reduction phase may prove difficult when implanting the DMC via the DAA. This renders it difficult to assess the leg length when the DMC is used through the DAA, thereby potentially increasing the risk of unacceptable leg length discrepancy (LLD). Then, we were led to raise a question: Does LLD occur more frequently in THA via the DAA with fluoroscopy using DMC than those using single mobility (SM)? This retrospective comparative study was performed to evaluate the differences in post-THA LLD between DAA with fluoroscopy using DMC and SM.

## Patients and methods

### Patients

The DMC has been available in Japan since 2013. At our university hospital, in patients of advanced age, we changed the main implant for primary THA from the SM cup to the DMC in 2013. From 2013 to the present, our indications for the use of the DMC were all elective surgeries for patients aged ≥70 years and those aged 65 to 69 years with a high risk of dislocation or medical complications associated with a short life expectancy. The DMC was generally not used for patients aged ≤64 years except patients with a femoral neck fracture due to bone fragility. In total, 386 THAs of all types were performed in our department from October 2011 to January 2015. In the period of this study, the DAA was used for primary THA by all surgeons in our department, with the exception of one surgeon who used the posterior approach. Among them, 60 hips of 58 patients operated by the DAA met the indication for use of a DMC as described above. The exclusion criteria for this study were osteoarthritis on the contralateral side, hip abduction or adduction of > 10° as shown on radiographs, rheumatoid arthritis, osteonecrosis, severe hip dysplasia (Crowe type III–IV), and a history of hip surgery. After exclusion against the criteria, 34 hips of 34 patients were analyzed.

Serving as controls in this study, the patients in the same periods, mainly before DMC introduction 2013, treated via the DAA using a polyethylene liner fixed to the metal cup (Single mobility cup), were initially included. Then, the 57 patients aged over 65 years (60 hips involved) were selected. In the 57 patients (60 hips), the same inclusion and exclusion criteria as aforementioned were applied. Finally, 31 hips of 31 patients were used as historical control group.

The DMC group is hereafter termed the DMC-DAA group, and the control group is referred to as the SM-DAA group.

No difference in the average age, sex ratio, body mass index, reason of THA was observed between the two groups (Table 1).

**Table 1:** Patient’s characteristics in the two groups

	Single-DAA	Dual-DAA	*p* value
Number of hips	31	34	
Age	73.9 ± 6.8	77.0 ± 6.7	0.07
Weight	54.6 ± 10.7	54.7 ± 10.3	0.93
BMI	22.8 ± 5.6	23.5 ± 4.3	0.59
Sex (% of females)	83.9	82.4	0.87
Etiology (OA / FNF / RA)	24 / 6 / 1	33 / 1 / 0	

*BMI* Body Mass Index, *OA* Osteoarthritis, *FNF* Femoral Neck Fracture, *RA* Rheumatoid Arthritis

### Implant details

The Trident HA (Stryker Orthopaedics, Mahwah, NJ, USA) (median, 48 mm; range, 44–54 mm) and Dual Mobility System (Stryker Orthopaedics) (median, 50 mm; range, 46–58 mm) were used as the cups in the SM-DAA and DMC-DAA groups, respectively. The Accolade TMZF (Stryker Orthopaedics) (127° neck angle) was employed as the stem in both groups. The diameter of the inner head was 32 mm in 31 hips in the SM-DAA group and the median outer diameter of the mobile polyethylene was 42 mm (range, 36–48 mm) in 34 hips in the DMC-DAA group.

### Operative procedure and intraoperative assessment of leg length

**Fig. 1:**
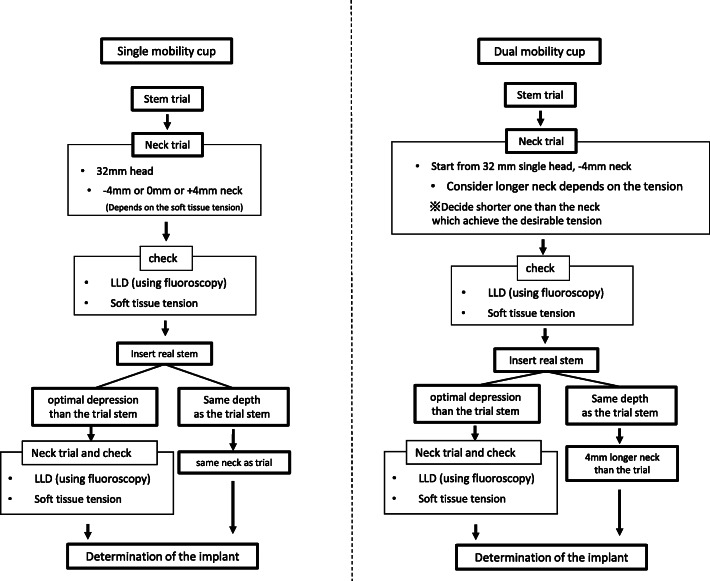
Operative protocol of reduction/dislocation phase using trial and leg length control

The DAA was performed as previously reported by using the distal part of the Smith-Petersen approach with the patient in the supine position on a standard table [16–18]. Intraoperative fluoroscopy was used for assessment of leg length [19]. We assessed the leg length by two different methods (Fig. 1). When the DMC is used through the DAA, intraoperative leg length assessment, including reduction or dislocation, using a trial implant is difficult because of high soft tissue tension. Once the trial DMC implant is reduced, it is almost impossible to dislocate it. Therefore, when the DMC was indicated, a 32-mm trial head of conventional SM and the shortest trial neck were used, and the LLD was assessed by comparison with the non-operative limb using intraoperative fluoroscopy. If the soft tissue tension was excessively great with the 32-mm trial head of conventional SM and the shortest trial neck, a 28-mm trial head of conventional SM and the shortest trial neck were used and reduced, and the LLD was assessed by using intraoperative fluoroscopy. If the soft tissue was not excessively tight with 32-mm trial head of conventional SM and the shortest trial neck, a trial neck one size longer than the shortest trial neck or a polyethylene liner one size smaller than the actual size of the DMC polyethylene liner was used for fluoroscopic assessment of the LLD during the operation.

**Fig. 2:**
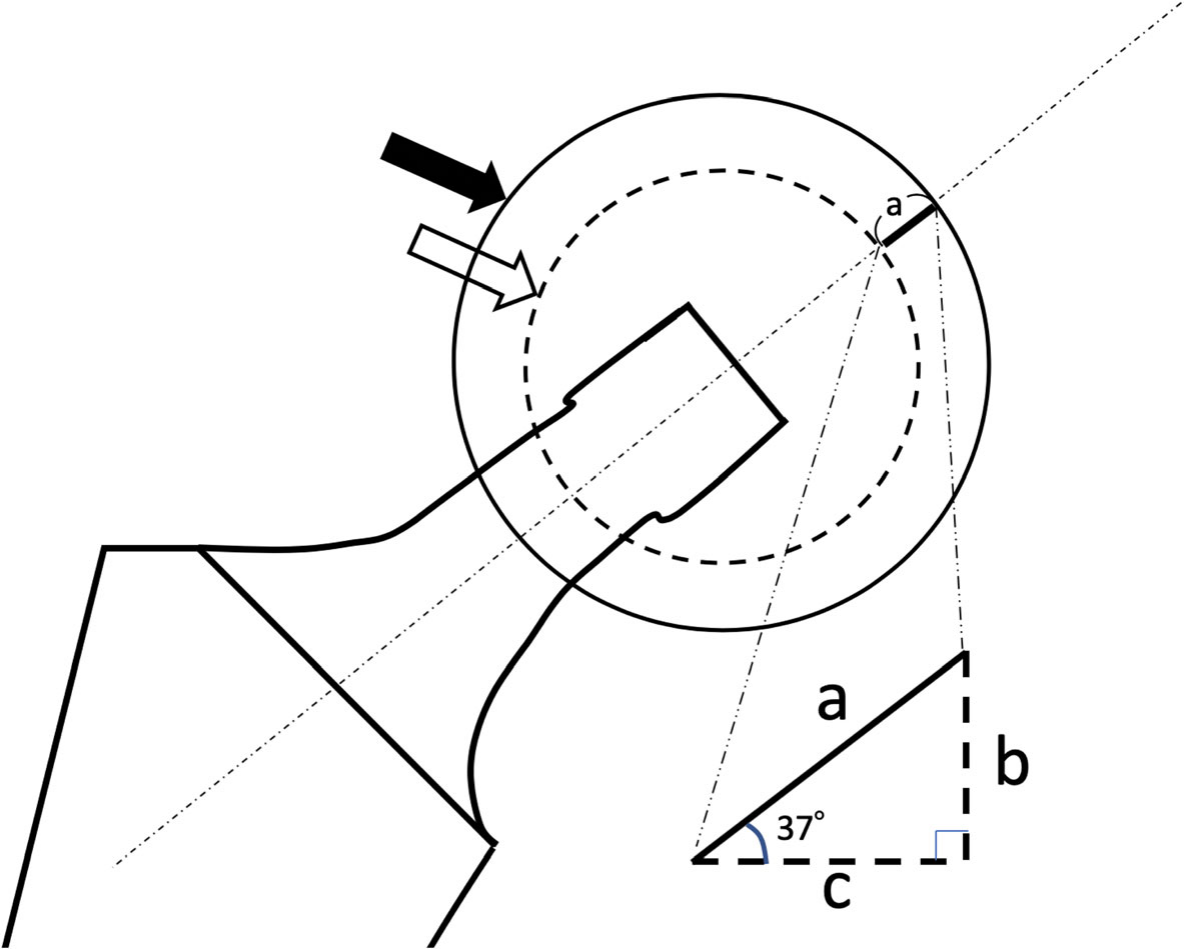
Image of extended length between 32 mm trial head and mobile polyethylene liner used in dual mobility system. Black arrow indicates mobile polyethylene liner, and white arrow indicates 32 mm trial head. We used stem angle only 127°, extended length along to body axis (**b**) is calculated by extended length along to neck axis (**a**) multiplying sin 37°. Extended length along to offset axis (**c**) is calculated by extended length along to neck axis (**a**) multiplying cos 37

Several variations of the outer diameter of X3 polyethylene insert for dual mobility cup were used against its corresponding Trident HA PSL CUP. The length of neck axis in the final dual mobility cup differs from those in 32-mm or 28-mm trial head (Fig. 2). The calculation chart shows lengths corresponding to neck, body, and offset axis using dual mobility system and a 32-mm trial head is summarized in Table 2. The amount of intraoperative leg extension was fluoroscopically estimated by using a 32- or 28-mm trial head size. In the SM-DAA group, the head and neck of actual size were used for the trial reduction and assessment of the LLD.

**Table 2:** The calculation chart showing length along to neck, body, and offset axis using dual mobility system and 32 mm trial head

		If the single mobility 32 mm trial head is used.		
Implanted Cup diameter (mm)	Dual Mobility Polyethylene outer diameter (mm)	Extended length to neck axis (mm)	Extended length to body axis (mm)	Extended length to offset axis (mm)
46 or 48	38	3	1.8	2.3
50 or 52	42	5	3	4
54 or 56	46	7	4.2	5.6
58 or 60	48	8	4.8	6.3

### Radiographic measurements

**Fig. 3:**
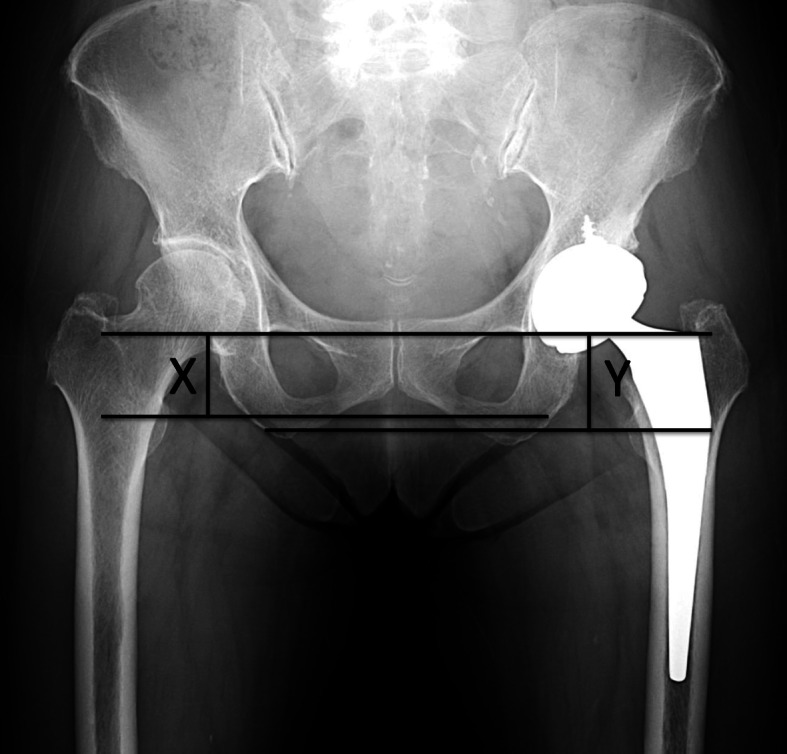
The distance between a line passing through the lower edge of the teardrop to the tip of the lesser trochanter of the operated (X) and the non-operated hip (Y) were measured. The difference of the each side (X-Y) was defined as the LLD

Radiographic parameters were measured on the immediate postoperative anteroposterior radiographs of the hips with both legs internally rotated to 15°. A horizontal axis was constructed on the radiograph at the distal-most part of the teardrop. Horizontal lines parallel to this axis were then constructed at the medial-most point of the lesser trochanter. The LLD was defined as the difference in the distance from the teardrop to the medial-most point of the lesser trochanter between the operative and non-operative sides (Fig. 3). If the operative side was longer than the non-operative side, the LLD was expressed as a positive value. If the operative side was shorter than the non-operative side, the LLD was expressed as a negative value. The LLD was assessed by its absolute value, which was categorized into three groups: < 5 mm, 5 to 10 mm, and > 10 mm.

### Statistical analysis

Baseline characteristics were expressed as mean ± standard deviation. Student’s independent-samples *t* test or the Mann-Whitney test was used for continuous variables, and the chi-square test was employed for dichotomous variables. A *P* value of < 0.05 was considered statistically significant, and all tests were two-sided. Data were statistically analyzed using IBM SPSS Statistics for Macintosh, Version 22.0 (IBM Corp., Armonk, NY, USA).

## Results

The mean LLD in the DMC-DAA group and SM-DAA group was 0.68 ± 7.7 mm (range, − 6.8 - 19.0) and 0.8 ± 5.5 mm (range, − 15.3-15.2), respectively, with no significant difference (*p* = 0.50). The absolute value of the LLD in the DMC-DAA group and SM-DAA group was 6.33 ± 4.1 mm (range, 0.2–19.0) and 5.91 ± 5.5 (range, 0.6–15.3), respectively, with no significant difference (*p* = 0.68). Comparison of the absolute value of the LLD in the DMC-DAA group showed an LLD of < 5 mm in 13 of 34 patients, 5 to 10 mm in 16 of 34 patients, and > 10 mm in 5 of 34 patients. The same comparison in the SM-DAA group exhibited an LLD of < 5 mm in 17 of 31 patients, 5 to 10 mm in 10 of 31 patients, and > 10 mm in 4 of 31 patients. No significant differences were found (Table 3).

**Table 3:** Difference of Leg length discrepancy between two groups

	Single: *n* = 31	Dual: *n* = 34	*p *value
Average difference (mm)			
LLD	0.80	0.68	0.50
Absolute value of LLD	5.91	6.33	0.68
Number of Patients: *n* = 65			
LLD < 5 mm: *n* = 30	17 (56.7%)	13 (43.3%)	
5 mm < LLD < 10 mm: *n* = 26	10 (38.4%)	16 (61.5%)	0.388
10 mm < LLD: *n* = 9	4 (44.4%)	5 (55.6%)	

*LLD* Leg Length Discrepancy

## Discussion

The dislocation and reduction procedure during THA with DMC via the DAA using a trial implant is difficult because of high soft tissue tension and the large polyethylene head [15]. Those limitations led us to ask the question: Does LLD occur more frequently in THA via the DAA with fluoroscopy using DMC than those using SM? However, our study demonstrated that the risk of LLD was no higher than that with an SM cup. As previously reported [11], we believe that the superiority of the DAA and DMC has a positive synergistic effect on resistance to dislocation without increasing the risk of complications such as LLD.

LLD is one of the major complications of THA related to lumber pain [20] [21], gait disorder [22], and low patients' satisfaction [23]. In the past decade, LLD is a second reason of litigation after THA [24].

Freiberg mentioned the rate of severe LLD (> 15 mm) in patients with chronic lumber pain (11.7%) was higher than in those without lumber pain (2.2%). They reported that bending and torsional stress to lower intervertebral disc imposed by LLD caused lumber pain [20]. Beard et al. showed that patients with LLD of 10 mm or more had predominantly lower Oxford Hip Scores 1 and 3 year(s) after operation [25], and Fujita et al. revealed that patients with radiographic LLD 8 mm or more easily perceived LLD and discomfort than those with LLD less than 7 mm [26].

We believe that intraoperative fluoroscopy is a very useful tool, which helps decrease the incidence of complications, including LLD. Although the actual size of the trial implant was not used for assessment of the LLD in our operative protocol, the use of fluoroscopy with an undersized trial implant allowed for estimation of the LLD with the actual size of the final implant. Because the actual size of the trial implant was not used in our protocol, which was very difficult in the reduction and dislocation phase, intraoperative femoral fracture did not occur in our series. Many authors have demonstrated the usefulness of intraoperative fluoroscopy in DAA THA [19, 27, 28]. Kobayashi et al. [29] investigated the implant positioning in the first 80 consecutive cases of THA performed by 2 senior surgeons using the DAA with fluoroscopic assistance and compared them with previous 80 respective cases of THA performed by the same two surgeons using previous standard posterior approach. They concluded that the cup positioning accuracy was higher for the DAA with fluoroscopy than for the standard posterior approach without fluoroscopy [29].

Not only THA through DAA but combination use of DMC and DAA helps further lower the dislocation risk. Impingement of the neck and polyethylene liner or cup due to the change in the angle of the hip joint causes lateral translocation of the femoral head center, eventually resulting in dislocation. The DMC has a greater oscillation angle than a standard cup and has the advantage of an anti-dislocation effect. Additionally, DAA enables performance of THA without cutting muscles and tendons, which helps maintain adequate soft tissue tension. Homma et al. reported that postoperative dislocation rate was low through DAA in their series, and DM group had no dislocation case (DAA-DM; 0%, DAA-SM; 1.7%, *p* = 0.315). This is the only investigation which reported the dislocation rate of cases with DAA and THA, while the dislocation rate in the DM group with all approaches was 0.9% [11].

Although polyethylene wear and intraprosthetic dislocation are major disadvantages of the DMC, other minor complications must be prevented to improve the clinical outcomes. Prudhon et al. [30] reported the reasons for revision of DMC THA and mentioned a high tendency of “other” reasons for the DMC. The LLD is generally categorized into “other” reasons. Therefore, the potential risk of LLD when using the DMC warrants investigation. Batailler et al. [14] also reported that THA using DAA and DMC did not increase the risk of complications, implant malpositioning or LLD. The findings were consistent with our conclusion.

This study had some limitations. First, the LLD was measured on plain radiographs in the supine position. X-rays with flex contracture, in valgus/varus positions or at malrotation will lead to measurement errors. Although we excluded radiographs with hip abduction or adduction by > 10°, radiographs might still be less accurate than computed tomography scans. In general, however, the LLD was measured on plain radiographs. Thus, we believe that our method was adequately validated. Second, the functional LLD in standing position could be more associated with patients’ satisfaction. X-ray involving the whole leg length in standing position was not taken in all patients, and the best postoperative leg length in patients with coronal pelvic obliquity in standing position was not clear, as the amount of postoperative pelvic obliquity change varies with different patients [31]. After all, in most cases of this study, the aim was to make the anatomical leg length identical on the both sides in supine position using intraoperative fluoroscopy. Third, although the surgeries were performed by multiple surgeons, all the surgeons were evaluated by the same processes when they adjusted both leg length using fluoroscopy. Therefore, the operative data can be comparable.

Finally, the study did not include a large number of patients. However, the size of the SM-DAA group as a historical control group was limited, and the number of patients with a normal contralateral hip was also limited. However, the purpose of this study was to compare difference in the LLD between DMC-DAA and SM-DAA. Thus the number of patients in this study was adequate for this purpose.

## Conclusion

The DMC-DAA group did not show a greater LLD than the SM-DAA group. No difference in the LLD, which has been thought to be a disadvantage of DMC-DAA, was found in this study.

## Data Availability

Not applicable.

## Abbreviations

THA: Total hip arthroplasty
DMC: Dual mobility cup
DAA: Direct anterior approach
LLD: Leg length discrepancy
